# Aggressive Angiomyxoma in Pregnancy: A Rare Condition, a Common Misdiagnosis

**DOI:** 10.1155/2016/8539704

**Published:** 2016-05-05

**Authors:** J. Sampaio, I. Sarmento-Gonçalves, D. Ramada, T. Amaro, P. Tiago-Silva

**Affiliations:** ^1^Department of Gynaecology and Obstetrics, Hospital Pedro Hispano, 4464-513 Matosinhos, Portugal; ^2^Department of Pathology, Hospital Pedro Hispano, 4464-513 Matosinhos, Portugal

## Abstract

*Introduction*. Aggressive angiomyxoma is a rare mesenchymal neoplasm. Although benign in the majority of the cases, these neoplasms usually present a locally infiltrative nature and high rates of recurrence. Due to its rarity, misdiagnosis is a common problem.* Case Presentation*. We present one case of aggressive angiomyxoma in a 25-year-old pregnant woman. The patient presented with a large vaginal mass that was interpreted as a vaginal cyst. We performed surgical resection of the neoplasm and the correct diagnosis was only achieved after histological examination. With this case, we highlight the importance of considering this diagnosis in patients with genital and perineal masses of unknown origin and the impact of a correct preoperative diagnosis in patient's management and follow-up.* Conclusion*. Although aggressive angiomyxoma is rare, it should be considered in differential diagnosis of pelviperineal masses in young women. Its positivity to estrogen and progesterone receptors can justify enlargement and recurrence during pregnancy, although few cases are reported. Early recognition demands high index of suspicion for both gynaecologists and pathologists. Wide surgical excision with tumor free margins is the basis of curative treatment. Adjuvant therapy may be necessary for residual or recurrent tumors. Long-term follow-up is recommended.

## 1. Introduction

Aggressive angiomyxoma (AA) is a rare, acquired mesenchymal neoplasm with predilection for the pelvic and perineal regions, first described in 1983 by Steeper and Rosai. It is more frequent in young women with a female to male ratio of 6 : 1 and usually occurs around the third or fourth decades of life [1, 2]. Although benign, the term “aggressive” emphasizes the frequent local recurrence and its infiltrative nature [3]. Due to its rarity the misdiagnosis rate was reported to be 80% [1, 2].

We present one case of vaginal aggressive angiomyxoma in a pregnant woman, whose initial diagnosis was different from the operative diagnosis, highlighting the importance of high suspicion indexes by both gynaecologists and pathologists.

## 2. Case Presentation

**Figure 1:**
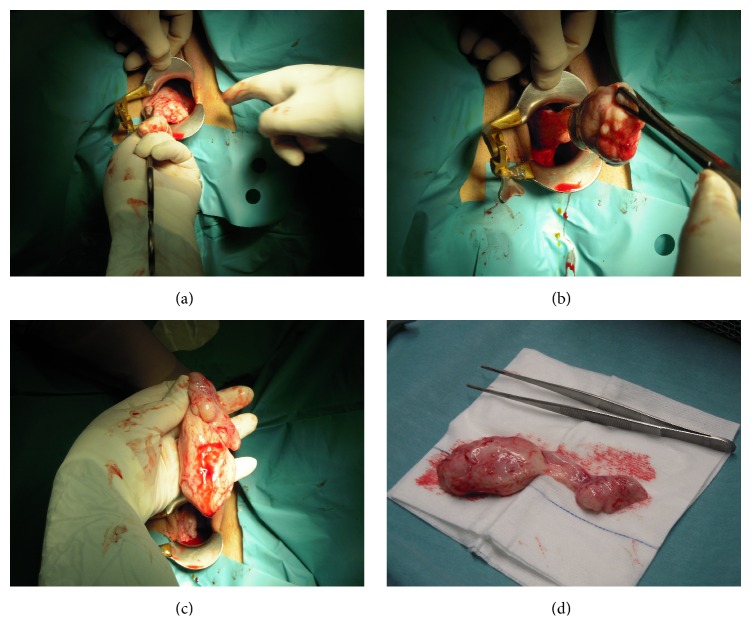
(a) Gelatinous mass arising from lateral vaginal wall. (b)-(c) Surgical excision of the mass. (d) Excised pedunculated mass being about 12 cm long.

A 25-year-old healthy woman, 9 weeks' pregnant, presented to our institution with a progressive swelling in the vagina with exponential growth in the latest weeks. The patient also referred to dyspareunia and coital bleeding. Her gynaecological history included excision of a similar mass four years ago in another hospital, without a histological diagnosis. Clinical examination revealed a glistening, gelatinous, nontender vaginal mass. The tumor arose from the right lateral vaginal fornix and it was a large, well-circumscribed, pedunculated mass (Figure 1(a)). Ultrasound examination showed a large mass being 11 cm long and with intermediate echogenicity. A diagnosis of probable vaginal wall cyst was made.

The patient was submitted to a complete surgical excision of the mass during the 13th week of gestation (Figures 1(b)–1(d)), which revealed its solid nature and the extension to the right paravaginal tissues. The procedure was performed under general anesthesia.

**Figure 2:**
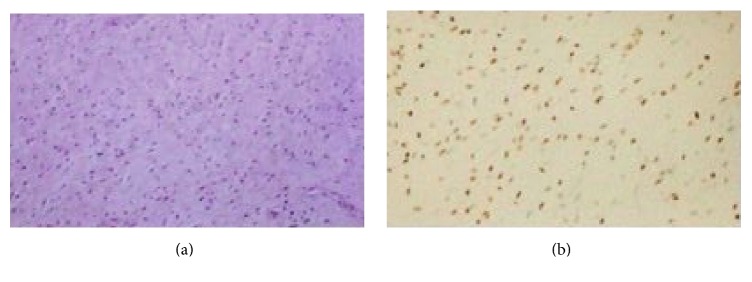
(a) HE stain. Aggressive angiomyxoma: evenly distributed delicate spindle cells grow in a myxoid matrix. (b) Immunohistochemical stains for smooth muscle actin, estrogen, and progesterone receptors are positive.

At macroscopic examination, the tumor measured 12 × 5 cm with a lobulated appearance, solid and rubbery. The cut surface was whitish and homogeneous. Microscopy revealed a paucicellular neoplasm composed of round and stellate cells with ill-defined cytoplasm and bland cytomorphology in a loose myxoid stroma. There was a prominent population of thick and thin-walled vessels, with no mitotic figures (Figure 2(a)). Immunohistochemistry was positive for vimentin, smooth muscle actin, estrogen, and progesterone receptors (Figure 2(b)). The neoplasm was negative for S100 protein, epithelial membrane antigen (EMA), and CD34, aspects consistent with AA.

In spite of not having access to the definitive diagnosis of the vaginal mass excised a few years ago, we believe that this might be a case of recurrent AA.

The postoperative follow-up was uneventful and pregnancy was held to term without complications. The patient has a three-year follow-up free of disease.

## 3. Discussion

AA is an uncommon mesenchymal neoplasm that occurs predominantly in young female adults in the pelvic and perineal regions [3]. This tumor has also been reported to develop in the retroperitoneum, urinary bladder, vulva, vagina, scrotum, and buttocks and it usually manifests as a polypoid or cystic like lesion or as an ill-defined swelling in the pelvic region [1]. On clinical examination it is usually mistaken for vulvar abcess, Bartholyn's cyst, Gartner's duct cyst, vaginal prolapse, pelvic floor hernia, vaginal mass or polyp, and obturatory or levator hernia [1]. As AA is a very rare cause of perineal mass, misdiagnosing will always be a problem and correct diagnosis is often suggested only after histological examination [4, 5]. AA is regarded as one of the mainstream soft-tissue myxomas. Angiomyofibroblastoma, cellular angiofibroma, superficial myofibroblastoma, and fibroepithelial polyps are other conditions, which can occur predominantly over the perineum and may be confused with AA [3].

Clinically AA typically presents a slow and insidious growth and is often asymptomatic [6]. The size of AA can fluctuate widely, but most of them are more than 10 cm long at diagnosis. It often presents as a large lesion that fills much of the pelvis, displacing the pelvic structures rather than directly invading them [7]. Although benign, the term “aggressive” emphasizes the frequent local recurrence and its infiltrative nature [3]. To our knowledge, there are only three cases of metastasised AA described in literature [8–10].

Grossly AA is typically a poorly circumscribed lesion with a gelatinous, myxoid, or fibrous consistency [7]. Microscopically, the tumor is composed of spindle and stellate-shaped cells in a myxoid matrix composed of delicate wavy collagen fibrils. There is also a prominent accompanying vascular component with vessels of different sizes [1, 5, 11–13]. Most of these tumors show reactivity for desmin, smooth muscle actin (SMA), muscle specific actin, vimentin, CD34, estrogen, and progestin receptors in immunohistochemical analyses. S100 protein is invariably negative [1, 7, 12]. The great majority of these neoplasms show estrogen and progesterone receptor positivity suggesting that AA is a hormone dependent tumor as rapid growth and recurrence have been observed during pregnancy [3, 13].

The pathogenesis of these tumors is unclear. Recently an association between chromosomal 12 translocation (12q13-15) and consequent aberrant expression of the high-mobility group protein isoform 1-C (HMG1-C) has been suggested [3, 12].

Ultrasound evaluation of these lesions usually shows a hypoechoic mass that can have a cystic appearance. CT scans appearances are variable, but AA usually manifests as a well-defined homogenous hypodense mass relative to muscle, a hypoattenuating solid mass with swirling internal pattern with contrast or as a cystic mass with solid components [5]. The most useful information about AAs is most likely provided by MRI, which offers the best resolution of AAs and their relationship with surrounding soft tissues associated with lack of ionizing radiation [2, 14].

It is consensual that wide surgical excision with free margins is the treatment of choice for AA. However, complete excision is difficult in some cases [2]. Hormonal treatment with raloxifene, tamoxifen, and gonadotropin-releasing hormone analogs (GnRH agonists) has been suggested as a primary treatment for small tumors and adjuvant therapy for residual disease and even for treatment of recurrence [12]. Aromatase inhibitors have also been tried in postmenopausal women prior to surgical approach [12]. These agents may also be useful in reducing the size of the lesion, especially when recurrent, allowing less radical surgical approaches [7]. Another treatment modality described in literature is angiographic embolization in attempt to devascularize the tumor, facilitating further resection of the neoplasm [13].

Due to low mitotic activity, it is unlikely that radiation or chemotherapy will be a useful adjunct to primary surgical treatment [1, 12]. However, radiotherapy should be considered in patients who do not respond to embolization or hormonal treatment, who experience persistent complaints, and in whom major, mutilating surgery is needed [13].

The local recurrence rate for AA is about 30–40% and can occur after 10–15 years of primary excision (2 months to 15 years) [4, 15]. For this reason long-term follow-up with MRI or CT scan has been recommended [4, 12]. Some authors have suggested the use of repeated surgery, radiotherapy, and hormonal therapy in cases of recurrence. However, there is no evidence of superiority of one modality over the others [12]. Metastases are very rare and overall prognosis is good [3].

In conclusion, although AA is a rare mesodermal tumor, it should be considered in differential diagnosis of pelvic and perineal mass in young female patients. Its positivity to estrogen and progesterone receptors can justify enlargement and recurrence of these tumors during pregnancy, although few cases are reported. Early recognition demands high index of suspicion for both gynaecologists and pathologists. In the case we describe, angiomyxoma was not considered as a differential diagnosis and so we did not perform any additional studies as biopsy of the lesion or MRI. If clinically suspected preoperatively, CT scan or MRI should be done to help in planning the surgery.

Wide surgical excision with tumor free margins is the basis of curative treatment of AA. We believe that high recurrence rates are due to an initial misdiagnosis and insufficient surgical excision. Adjuvant therapy may be necessary for residual or recurrent tumors. The hormone dependent characteristics suggest that hormonal treatment may be valuable.

Long-term follow-up with MRI examination is recommended because the tumor may be indiscernible at pelvic and ultrasound examination.
